# Tryptophanyl tRNA Synthetase from Human Macrophages Infected by *Porphyromonas gingivalis* Induces a Proinflammatory Response Associated with Atherosclerosis

**DOI:** 10.3390/pathogens10121648

**Published:** 2021-12-20

**Authors:** Minoru Sasaki, Yu Shimoyama, Yoshitoyo Kodama, Taichi Ishikawa

**Affiliations:** Department of Microbiology, Division of Molecular Microbiology, Iwate Medical University, Morioka 028-3694, Japan; yushimo@iwate-med.ac.jp (Y.S.); microbio@catv-mic.ne.jp (Y.K.); tishikaw@iwate-med.ac.jp (T.I.)

**Keywords:** *Porphyromonas gingivalis*, aminoacyl-tRNA synthetases, macrophages, HUVECs, periodontal diseases, atherosclerosis

## Abstract

*Porphyromonas gingivalis* is the most common microorganism associated with adult periodontal disease, causing inflammation around the subgingival lesion. In this study, we investigated tryptophanyl tRNA synthase (WRS) production by THP-1 cells infected with *P. gingivalis*. Cytokine production, leukocyte adhesion molecules, and low-density lipoprotein receptor (LDLR) expressions in cultured cells were examined. WRS was detected in THP-1 cell culture supernatants stimulated with *P. gingivalis* from 1 to 24 h, and apparent production was observed after 4 h. No change in WRS mRNA expression was observed from 1 to 6 h in THP-1 cells, whereas its expression was significantly increased 12 h after stimulation with *P. gingivalis*. Lactate dehydrogenase (LDH) activity was observed from 4 to 24 h. The TNF-α, IL-6, IL-8, and CXCL2 levels of THP-1 cells were upregulated after treatment with recombinant WRS (rWRS) and were significantly reduced when THP-1 cells were treated with C29. The MCP-1, ICAM-1, and VCAM-1 levels in human umbilical vein endothelial cells were upregulated following treatment with rWRS, and TAK242 suppressed these effects. Additionally, unmodified LDLR, macrophage scavenger receptor A, and lectin-like oxidized LDLRs were upregulated in THP-1 cells treated with rWRS. These results suggest that WRS from macrophages infected with *P. gingivalis* is associated with atherosclerosis.

## 1. Introduction

*Porphyromonasgingivalis*, which inhabits the subgingival plaque biofilm, is a gram-negative anaerobic rod bacterium and is the most common bacterium associated with adult periodontal disease [1,2]. Chronic periodontal disease causes an immune response in the host, both innate and adaptive, in which cells are recruited from the systemic circulation [3]. As a result, many immune cells or inflammatory cytokines induce severe inflammation of subgingival tissues. These biological reactions in the host as a result of *P. gingivalis* infection have also been suggested as a potential risk factor for several systemic diseases, such as diabetes, premature birth, heart disease, and atherosclerosis, as well as local lesions [4,5,6,7].

*P. gingivalis* possesses many virulence factors that can cause tissue destruction or induce inflammation via other mediators [8,9]. The biological activities and role of *P. gingivalis* lipopolysaccharide (Pg-LPS) have been extensively investigated. Pg-LPS can stimulate the expression of IL-1β, TNF-α, IL-6, and the receptor activator of NF-κB ligand by activating the innate immune response of nonimmune cells such as gingival fibroblasts [10,11,12,13]. In addition, *P. gingivalis* pili play an important role in the adhesion properties to host molecules and oral substrates. The fimbriae are recognized by receptors on epithelial, endothelial, and immune cells, resulting in cell activation and cytokine and adhesion molecule production [14,15,16]. Gingipain is an important factor in the pathogenesis of periodontitis for degrading gingival epithelial cell adhesion molecules and disrupting the epithelial barrier [17,18]. Gingipain is also involved in the regulation of the immune response and the production of immune mediators in various cells [19,20]. So far, the pathogenicity of *P. gingivalis* has been investigated, mostly based on its bacterial components.

Aminoacyl-tRNA synthetase (ARS) is an enzyme that binds amino acids, transfers RNA, and synthesizes aminoacyl-tRNA, which is required for ribosomal protein synthesis. ARS also serves as a multifunctional moonlighting protein that performs additional cellular functions, both intracellularly and extracellularly. It has been reported that some ARSs are secreted by intact or necrotic cells and serve as cytokines [21,22,23,24]. Ahn et al. suggested that tryptophanyl tRNA synthetase (WRS) secreted by macrophages in the early stages of bacterial and viral infection induces cytokine production in host cells [25]. WRS has been suggested to be involved in host defense as a protective alert in the acute phase, the so-called risk detection, and rapid response. Although WRS functions as an innate immune factor, the continuous release of WRS can cause inflammation when microbial antigens stimulate host cells, for example, during chronic infection. The production of WRS by host cells infected with periodontal bacteria can cause local inflammation of subgingival tissues and systemic disease as the bacteria migrate to various sites in the body. However, no studies have investigated the production of WRS by host cells infected with periodontal bacteria.

In this study, we investigated the responsiveness of WRS production by macrophages infected with *P. gingivalis*. Then, we examined the effects of WRS on the expression of inflammatory cytokines, chemokines, and leukocyte adhesion molecules in human umbilical vein endothelial cells (HUVECs) or THP-1 cells. Macrophages actively take up unmodified low-density lipoprotein (LDL) and/or modified LDL, such as oxidized LDL (oxLDL) [26,27]. The uptake of unmodified LDL or oxLDL via the LDL receptor (LDLR) or scavenging receptors (SRs) by macrophages leads to the formation of mature lipid-laden macrophages called foam cells and is associated with atherothrombotic disease. Therefore, in this study, we investigated the expression of LDLR and SRs, macrophage SR class A (SR-A), CD36, and lectin-like oxLDL receptor-1 (LOX-1) in THP-1 cells treated with WRS. Thus, in this study, we investigated whether WRS, an intracellular multifunctional protein, can be related to systemic disease, especially atherosclerosis, following *P. gingivalis* infection.

## 2. Results

### 2.1. WRS Levels in THP-1 Cells Stimulated with P. gingivalis

**Figure 1:**
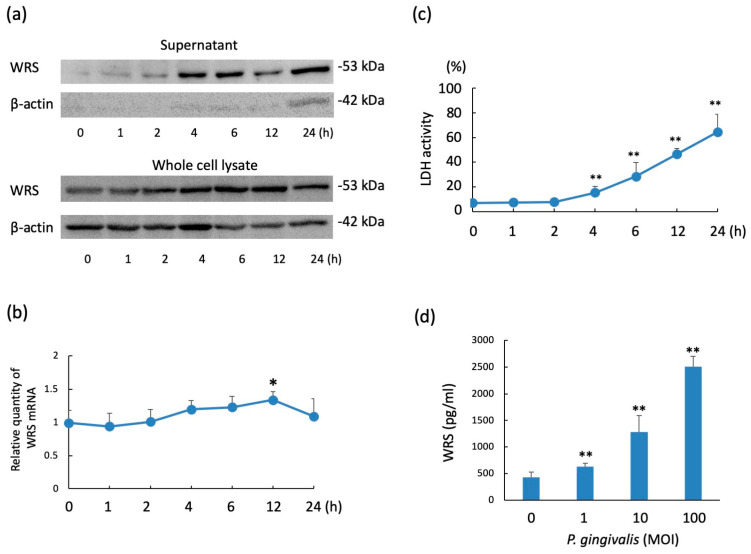
WRS expression levels in THP-1 cells following infection with *P. gingivalis*. (**a**) Detection of WRS in culture supernatants and whole cell lysate of THP-1 cells following *P. gingivalis* infection. The cells (1 × 10^5^/well) were stimulated with *P. gingivalis* (MOI: 10) at 37 °C for the indicated times in serum-free RPMI 1640 medium supplemented with protease inhibitor cocktail. WRS and β-actin in the culture supernatants or whole-cell lysates were detected using SDS-PAGE, followed by western blotting using anti-WRS and anti-β-actin antibodies, respectively. (**b**) WRS mRNA expression levels in THP-1 cells stimulated with *P. gingivalis* (MOI: 10) at 37 °C for the indicated times. The relative expressions are shown after normalization against GAPDH mRNA expression. (**c**) LDH activity of THP-1 cells (1 × 10^5^/well) after stimulation with *P. gingivalis* (MOI: 10) for the indicated times at 37 °C in serum-free RPMI 1640 medium supplemented with protease inhibitor cocktail. The LDH activity was expressed as follows (%): activity of the culture supernatant of *P. gingivalis* infected cells (1 × 10^5^ cells)/activity of culture supernatant of 1% Triton X100-treated cells (1 × 10^5^ cells). (**d**) WRS levels in the culture supernatants with the indicated doses (MOI: 1, 10, 50, and 100) were estimated using an enzyme-linked immunosorbent assay. Data are expressed as the mean ± standard deviation from four independent experiments, each performed in duplicates. Statistically significant differences; ** *p* < 0.01, * *p* < 0.05.

The levels of WRS in the culture supernatants of the confluent culture of THP-1 cells (1 × 10^5^ cells) stimulated with the multiplicity of infection (MOI) 10 of *P. gingivalis* (1 × 10^6^ CFU) are shown in Figure 1a. WRS was detected from 1 to 24 h, and apparent WRS production was observed after 4 h. The WRS level in the whole cell lysate was detected constantly from 0 to 24 h following *P. gingivalis* stimulation. β-actin was detected in the supernatants at a very low level following *P. gingivalis* stimulation and was detected at 24 h. While no change in WRS mRNA expression was observed from 1 to 6 h in THP-1 cells, WRS mRNA expression was significantly increased 12 h after stimulation with *P. gingivalis* (Figure 1b). Lactate dehydrogenase (LDH) activity was observed from 4 to 24 h (Figure 1c). These results suggest that WRS in the culture supernatant was secreted by THP-1 cells at an early stage following infection (1~2 h). Conversely, WRS detected in the supernatant from 4 to 24 h could have leaked from THP-1 cells because of the partial destruction of the cells following *P. gingivalis* infection. The detection of WRS in THP-1 cells infected with *P. gingivalis* was significantly dose-dependent at a dose from MOI 1 to 100 (Figure 1d).

### 2.2. TNF-α and IL-8 Expressions in THP-1 Cells Treated with Recombinant WRS (rWRS)

**Figure 2:**
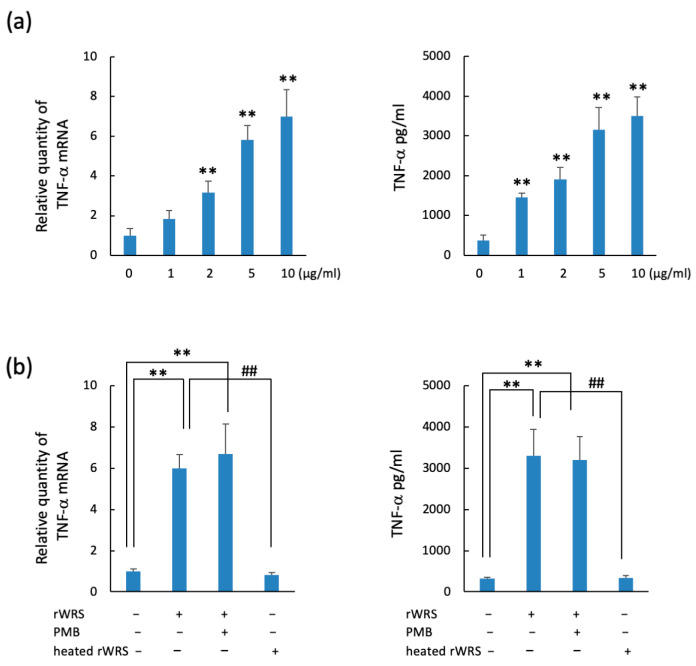
TNF-α expression in THP-1 cells treated with rWRS. (**a**) THP-1 cells were treated with the indicated doses of rWRS in serum-free RPMI 1640 medium at 37 °C for 2 h. The cells were harvested after incubation, and RNA was extracted using the RNeasy Mini Kit, followed by the preparation of complementary DNA. We determined the expression level of TNF-α mRNA using a quantitative real-time reverse-transcription polymerase chain reaction. Enzyme-linked immunosorbent assay was performed to estimate the protein level after treatment of THP-1 cells with rWRS for 24 h. (**b**) The effects of WRS treated with 10 µg/mL PMB at room temperature for 1 h or heated at 15 min at 100 °C on the expression of TNF-α were investigated. Data are expressed as the mean ± standard deviation from three independent experiments, each performed in duplicates. Statistically significant differences; ** *p* < 0.01, ^##^ *p* < 0.01.

**Figure 3:**
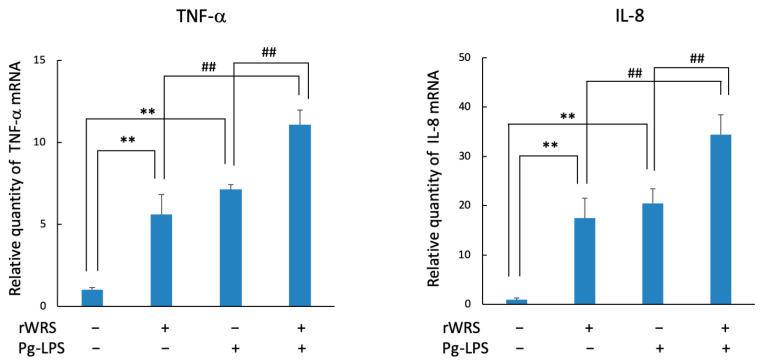
Additive expressions of TNF-α and IL-8 mRNA in THP-1 cells treated with rWRS and/or stimulated with Pg-LPS. THP-1 cells were incubated with rWRS (5 µg/mL) and/or Pg-LPS (10 µg/mL) in serum-free RPMI 1640 medium at 37 °C for 2 h. The mRNA expression levels of TNF-α and IL-8 were determined using a quantitative real-time reverse-transcription polymerase chain reaction. Data are expressed as the mean ± standard deviation from three independent experiments, each performed in duplicates. Statistically significant differences; ** *p* < 0.01, or ^##^ *p* < 0.01.

The TNF-α mRNA and protein levels of THP-1 cells treated with rWRS were upregulated (Figure 2a). In addition, the mRNA and protein expressions were enhanced in a dose-dependent manner. Although the cytokine upregulation of rWRS treated with polymyxin B (PMB) (10 µg/mL) was similar to that of untreated rWRS, it declined following the heating of rWRS at 100 °C for 15 min (Figure 2b). These results indicate that the upregulation is induced by rWRS itself as opposed to LPS present in the sample. Furthermore, the expression levels of TNF-α and IL-8 following rWRS treatment were similar to those when THP-1 cells were stimulated with Pg-LPS. In addition, the upregulation of these cytokines additively increased when THP-1 cells were stimulated by both stimulants (Figure 3). These findings suggest that WRS may further exacerbate the inflammatory response of bacterial cell components.

### 2.3. Cytokine Expression in THP-1 Cells Treated with rWRS and the Effect of the Inhibitor of TLR Signaling

**Figure 4:**
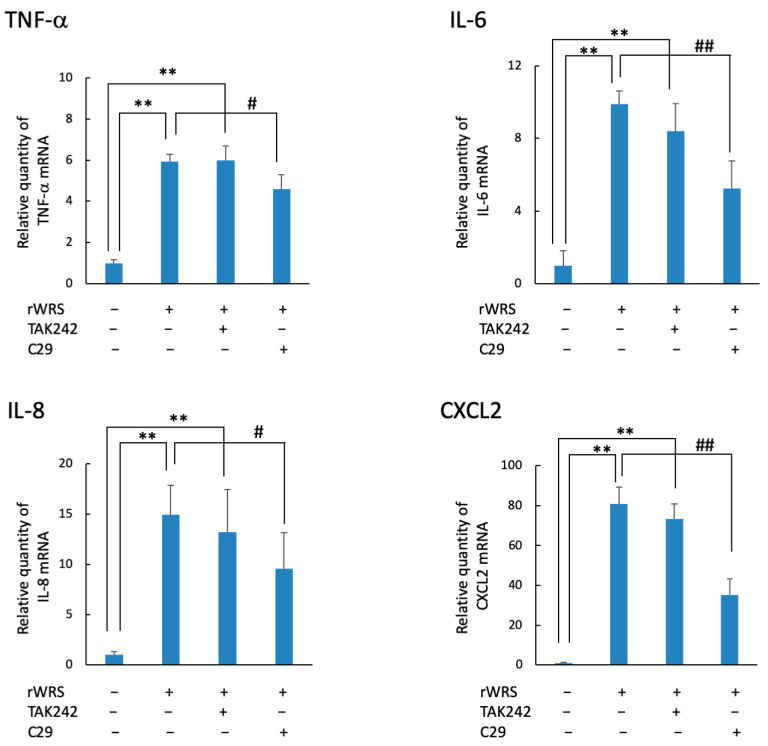
Effect of the TLR inhibitors TAK242 or C29 on the expression of cytokines and chemokines. THP-1 cells pretreated with or without TAK242 (1 µM) or C29 (50 µM) for 30 min in serum-free RPMI 1640 medium were incubated with 5 µg/mL rWRS at 37 °C for 2 h. The mRNA expressions of TNF-α, IL-6, IL-8, and CXCL2 were determined using a quantitative real-time reverse-transcription polymerase chain reaction. Data are expressed as the mean ± standard deviation from four independent experiments, each performed in duplicates. Statistically significant differences; ** *p* < 0.01, or ^##^ *p* < 0.01, ^#^ *p* < 0.05.

The mRNA levels of TNF-α, IL-6, IL-8, and CXCL2 in THP-1 cells treated with rWRS are shown in Figure 4. The mRNA expressions of these cytokines or chemokines were induced by rWRS, and they were significantly reduced when THP-1 cells were pretreated with C29, an inhibitor of Toll-like receptor (TLR)-2 signaling. Conversely, cells treated with TAK242, an inhibitor of TLR-4 signaling, showed similar expression levels as those stimulated with rWRS alone.

### 2.4. Chemokine and Adhesion Molecule Expressions in HUVECs Treated with rWRS

**Figure 5:**
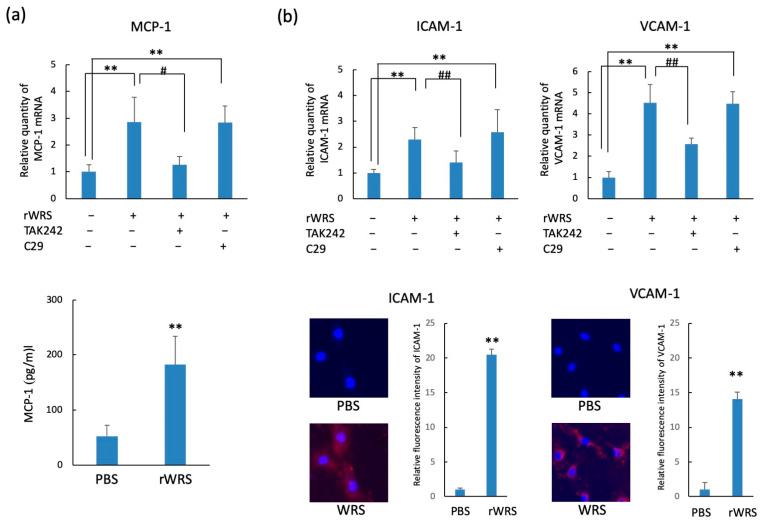
MCP-1 and leukocyte adhesion molecule expressions in HUVECs treated with rWRS. (**a**) HUVECs pretreated with or without TAK242 (1 µM) or C29 (50 µM) for 30 min in serum-free HuMedia-EG2 medium were incubated with 5 µg/mL rWRS at 37 °C for 2 or 4 h. The mRNA expressions of MCP-1, ICAM-1, and VCAM-1 were determined using a quantitative real-time reverse-transcription polymerase chain reaction. An enzyme-linked immunosorbent assay was performed to estimate the MCP-1 level in the culture supernatant. (**b**) The expressions of adhesion molecules in HUVECs treated with rWRS were analyzed using immunofluorescence. After 12 h of incubation at 37 °C, HUVECs treated with rWRS were fixed with paraformaldehyde. After washing the cells with phosphate-buffered saline (PBS) and then blocking them with 1% fetal calf serum, we treated them with monoclonal anti-ICAM-1 antibodies or anti-VCAM-1 antibodies overnight at 4 °C. After washing the cells with PBS, we treated them with Alexa Fluor 594-conjugated goat anti-rabbit immunoglobulin G and stained the nuclei with DAPI. Data are expressed as the mean ± standard deviation from four independent experiments, each performed in duplicates. Statistically significant differences; ** *p* < 0.01, or ^##^ *p* < 0.01, ^#^ *p* < 0.05.

The protein levels and mRNA expressions of MCP-1, ICAM-1, and VCAM-1 in HUVECs stimulated with rWRS are shown in Figure 5. The mRNA expressions of MCP-1, ICAM-1, and VCAM-1 in HUVECs were upregulated following treatment with rWRS. However, these enhancements were exclusively reduced by TAK242, an inhibitor of TLR-4. In addition, by performing enzyme-linked immunosorbent assay and immunostaining using anti-ICAM-1 and anti-VCAM-1 antibodies, we determined that the protein levels of MCP-1 and the adhesion molecules ICAM-1 and VCAM-1 in HUVECs were significantly upregulated following treatment with rWRS.

### 2.5. Effect of rWRS on Native LDLR and oxLDLR Gene Expressions in THP-1 Cells

**Figure 6:**
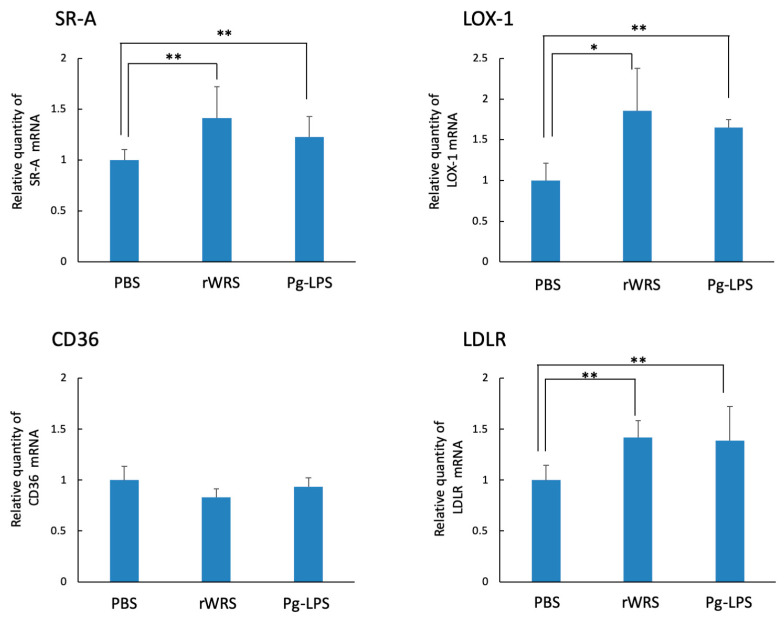
LDL receptor gene expression in THP-1 cells. The culture medium used for the differentiation of THP-1 cells (phorbol 12-myristate 13-acetate) was changed after 3 d to serum-free RPMI 1640. The THP-1 cells were further incubated in the medium for 4 h at 37 °C. Then, the THP-1 cells were treated with rWRS or Pg-LPS for 3 h at 37 °C. The quantitative real-time reverse-transcription polymerase chain reaction was performed to determine the gene expressions of SR-A, LOX-1, CD36, and LDLR. GAPDH mRNA was used as an internal control. Data are expressed as the mean ± standard deviation from four independent experiments, each performed in duplicates. Statistically significant differences; ** *p* < 0.01, * *p* < 0.05.

Each stimulant significantly induced the expressions of SR-A, LOX-1, and LDLR in THP-1 cells treated with rWRS or Pg-LPS, whereas the expression of the CD36 gene showed no change in rWRS- or Pg-LPS-treated THP-1 cells (Figure 6).

## 3. Discussion

In recent years, many investigators have suggested that oral bacteria are associated with systemic diseases [28,29]. *P. gingivalis*, which is mainly distributed in the oral cavity, is most commonly associated with chronic periodontitis and has been suggested to be associated with cardiovascular diseases, diabetes, premature birth, rheumatism, and myocardial infarction. *P. gingivalis* DNA is often detected in various body sites, such as plaques in thrombi [30,31]. This suggests that *P. gingivalis* hematogenously invades from the oral lesion and reaches the vascular lesion. Among systemic diseases, atherosclerosis is the most common cause of death and disability, and it induces the development of myocardial infarction [32,33,34]. Therefore, we focused on the association of *P. gingivalis* with atherosclerosis, which induces the development of myocardial infarction. Thus far, attention has been focused on the pathogenic mechanism of *P. gingivalis*, such as LPS or pili, which induce host inflammatory responses [11]. In this study, we investigated the production of WRS by cells infected with *P. gingivalis* and the biological activity of WRS in host cells to clarify a new pathogenic mechanism of atherosclerosis caused by *P. gingivalis*.

ARS is an intracellular protein that is involved in protein synthesis; recent studies have identified it as a novel molecule that controls host immune responses against infections. Tyrosyl tRNA synthetase (YRS) is secreted extracellularly and is cleaved into two distinct cytokines, yielding an N-terminal catalytic domain (mini-YRS) possessing an IL-8-like cytokine via its Glu-Leu-Arg motif and a C-terminal endothelial monocyte-activating polypeptide II-like domain [23,24,35,36]. Furthermore, lysyl tRNA synthetase (KRS) is secreted from macrophages stimulated with *Salmonella* toxin or TNF-α. The secreted KRS was found to bind to the host receptor of monocytes and macrophages to induce immune responses via the ERK and p38 MAPK signaling pathways [37,38,39]. Ahn et al. suggested that WRS secreted by macrophages in the early stages of bacterial infection activate host cells through TLR-2 or TLR-4 signaling [25].

In this study, the presence of WRS, although minimal, in THP-1 cell culture medium within 2 h following *P. gingivalis* infection, suggests that WRS was secreted by THP-1 cells, similar to that reported by Ahn et al. [25], because LDH activity was not detected within 2 h. In contrast, WRS and LDH activity were detected in the medium after 4 h. This suggests that WRS may have been secreted into the medium due to the partial destruction of THP-1 cells following *P. gingivalis* infection. Thus, secretion and leakage are both involved in the production of WRS by THP-1 cells due to *P. gingivalis* infection. In addition, human rWRS induced the expression of inflammatory cytokines, such as TNF-α and IL-6, and chemokines, such as IL-8 and CXCL2, in THP-1 cells. Similarly, the treatment of HUVECs with rWRS induced the expression of MCP-1, ICAM-1, and VCAM-1 at the gene and protein levels. Moreover, the expression-inducing activity of cytokines and chemokines in THP-1 cells treated with rWRS was inhibited by C29, an inhibitor of TLR-2 signaling. Conversely, HUVEC-inducing activity was inhibited by TAK242, suggesting that TLR-4-mediated expression was upregulated. This may be due to the difference in TLR-2 and TLR-4 expression levels between THP-1 cells and HUVECs [40].

WRS secreted in the early stages of infection is mainly considered to protect against pathogen infection, but the continuous secretion of WRS by the host cell during *P. gingivalis* infection can be a potent inflammatory substance in the pathogenic lesion, such as damage-associated molecular patterns (DAMPs) [41,42,43,44,45]. Atherosclerosis is generally recognized as a chronic inflammatory disease characterized by the excessive recruitment of white blood cells (monocytes and T cells) to the inflammation site. That is, the increased expression of adhesion molecules and the secretion of inflammatory cytokines and chemokines in response to cardiovascular risk factors lead to the attachment, migration, and accumulation of leukocytes within atherosclerotic lesions [46,47]. Infection by *Chlamydia pneumoniae*, which is suggested to be one of the etiologic agents of atherosclerosis, stimulates the secretion of inflammatory cytokines (TNF-α, IFN-γ, IL-6, etc.) [48] and leukocyte adhesion molecules [49]. These cause T cells and monocytes to migrate trans-endothelially and differentiate into macrophages. Heat shock protein 60 (HSP60) in *C. pneumoniae* is a homolog of human HSP60, regarded as a DAMP, and is considered the etiologic agent of the inflammatory response in atherosclerosis [48,50]. As shown in this study, the production of inflammatory cytokines and chemokines by macrophages following rWRS treatment could induce inflammatory cell recruitment and inflammatory responses at the infection site. In addition, the induction of MCP-1 and the leukocyte adhesion molecule expression of HUVECs could promote macrophage accumulation and tissue infiltration from blood vessels. These results indicate that the behavior of rWRS is similar to that of *C. pneumoniae* HSP60.

Mature macrophages migrate into the endothelium and actively take up LDL and/or oxLDL through LDLR and/or SRs, leading to the formation of mature lipid-laden macrophages (foam cells). It has been suggested that LDLR expression is upregulated by inflammatory stress and allows macrophages to ingest considerable amounts of native LDL [51]. Macrophage SR-A is considered to be one of the main receptors involved in mediating the influx of oxLDL into cells [52]. LOX-1 was initially identified as a major SR for oxLDL [53]. LOX-1 overexpression has been observed in endothelial cells and macrophages in human atherosclerotic lesions [54]. Therefore, in this study, the mRNA expression of these receptors was examined in THP-1 cells treated with rWRS. rWRS and Pg-LPS significantly induced the expressions of LDLR, SR-A, and LOX-1. Maekawa et al. reported that the upregulation of LDLR by murine macrophages treated with Pg-LPS was suppressed by the nuclear factor LXR that controls LDLR expression [51]. Because LPS signals are transmitted via TLRs, WRS, which has an affinity for TLR, may upregulate LDLR via a similar mechanism. SR-A and LOX-1 were upregulated in macrophages by bacterial LPS or lipoteichoic acid via TLRs of host cells [55]. Therefore, the upregulation of these LDLRs by rWRS in this study could also depend in part on TLRs, i.e., TLR-2 or TLR-4.

Collectively, these results suggest that WRS secreted by host cells following *P. gingivalis* infection induces the expression of inflammatory cytokines and chemokines from macrophages, as well as the expression of chemokines and adhesion molecules in HUVECs, leading to the recruitment of inflammatory cells in blood vessels and inflammatory reactions. In addition, rWRS upregulated LDLR and oxLDLR expressions, which may result in the formation of foam cells. Therefore, the secretion of WRS from THP-1 cells following *P. gingivalis* infection is considered one of the new pathogenic mechanisms of periodontal bacteria in atherosclerosis. ARS has also been reported to be involved in several immunological diseases [38,56]. The elimination of unwanted physiological activities of extracellular multifunctional proteins, such as ARS and DAMPs, may be a new therapeutic strategy for various inflammatory diseases.

## 4. Materials and Methods

### 4.1. Bacterial Strains and Culture Conditions

*P. gingivalis* ATCC 33277 was cultured in ABCM broth (Eiken, Chemical Co., Ltd., Tokyo, Japan) supplemented with 5 µg/mL hemin and 1 µg/mL menadione at 37 °C for 48 h under anaerobic conditions.

### 4.2. Cells and Culture Conditions

We purchased HUVECs from the Health Science Research Bank (Osaka, Japan). The cells were maintained in HuMedia-EG2 medium (Kurabo Ind. Ltd., Osaka, Japan) supplemented with 10% fetal calf serum, 10 ng/mL fibroblast growth factor basic, and penicillin/streptomycin solution at 37 °C in an atmosphere containing 5% CO_2_. THP-1 cells (RCB 1189; Riken Cell Bank, Tsukuba, Japan) were maintained in RPMI 1640 medium (Thermo Fisher Scientific, Roskilde, Denmark) supplemented with 10% fetal calf serum and penicillin/streptomycin solution at 37 °C in an atmosphere containing 5% CO_2_. The THP-1 cells were differentiated using 200 nM phorbol 12-myristate 13-acetate (PMA, Sigma-Aldrich, St. Louis, MO, USA) for 3 days.

### 4.3. Detection of WRS in Culture Supernatants and Whole Cell Lysate of THP-1 Cells Infected with P. gingivalis

PMA-differentiated THP-1 cells were cultured in serum- and antibiotic-free RPMI media containing a protease inhibitor mixture (Nacalai Tesque, Kyoto, Japan). The cells were stimulated with *P. gingivalis* at the indicated times and doses. The detection of WRS in the culture supernatants and whole cell lysate of THP-1 cells infected with *P. gingivalis* was demonstrated using SDS-PAGE, followed by western blotting [57]. Affinity-purified polyclonal primary antibodies against WRS (Sigma-Aldrich) and β-actin (Proteintech Group, Inc., Rosemont, IL, USA) were reacted at 4 °C for 3 h. The membrane was then washed with PBS supplemented with 0.1% Tween 20 and incubated with a 1:2500 dilution of goat anti-rabbit IgG conjugated with horseradish peroxidase at 4 °C for 1 h. We used a Chemi-Lumi One Super Western Blotting Detection System (Nacalai Tesque, Kyoto, Japan) for detection. The WRS levels in the culture supernatants infected with the indicated doses of *P. gingivalis* were estimated using an enzyme-linked immunosorbent assay kit (MyBioSource, San Diego, CA, USA), following the manufacturer’s instructions. The expression of WRS mRNA in THP-1 cells stimulated with *P. gingivalis* (MOI: 10) at 37 °C for the indicated times was also determined. The relative expressions were presented after normalization against GAPDH mRNA expression. In some experiments, the culture supernatants of THP-1 cells stimulated with *P. gingivalis* (MOI: 10) were analyzed for LDH activity using the LDH assay kit (Takara Bio, Shiga, Japan). The LDH activity was expressed as follows (%): activity of culture supernatant of *P. gingivalis*-infected cells (1 × 10^5^ cells)/supernatant of 1% Triton X100-treated cells (1 × 10^5^ cells).

### 4.4. Construction of an Expression Vector for WRS Protein and Expression and Purification of rWRS

cDNA was prepared from purified RNA obtained from gingival epithelial cells, Ca9-22, using the RNeasy Mini Kit (QIAGEN, Copenhagen, Denmark) and reverse-transcribed into cDNA (Takara Bio). A DNA fragment encoding human WRS Met^1^–Gln^471^ was amplified by PCR using KOD-Plus-Neo DNA polymerase, cDNA as a template, and a set of primers (5′-GCTCGGTACCATGCCCAACAGTGAGCCC-3′ and 5′-TAATTAAGCTTCTACTGAAAGTCGAAGGACAGC-3′) designed based on the gene (NCBI accession no. XM_017021629). The PCR product was excised using *Kpn*I and *Hind*III and inserted into the *Kpn*I and *Hind*III site of pQE-80L (QIAGEN). The PCR products were phosphorylated and self-ligated to produce the expression plasmids. The construct was ascertained with DNA sequencing. LPS-free *Escherichia coli* cells (ClearColi™ BL21 [DE3]; COSMO Bio, Tokyo, Japan) carrying an expression plasmid were cultured overnight in Luria-Bertani broth supplemented with 50 µg/mL kanamycin at 30 °C. Recombinant protein expression was induced by 0.2 mM isopropyl β-d-1-thiogalactopyranoside at 30 °C for 3 h and purified from cells via TALON affinity chromatography [58].

### 4.5. Cytokine, Chemokine, and Leukocyte Adhesion Molecule Expression

THP-1 cells treated with rWRS (5 µg/mL) or Pg-LPS (Sigma-Aldrich; 10 µg/mL) were incubated at 37 °C for 2 h in serum-free RPMI 1640 medium. The cells were harvested, and RNA was extracted using the RNeasy Mini Kit (QIAGEN) following the manufacturer’s instructions. Then, cDNA was prepared using the PrimeScript RT Reagent Kit (Takara Bio). We performed RT-qPCR to determine the expression levels of TNF-α, IL-6, IL-8, and CXCL2. In some experiments, THP-1 cells or HUVECs were treated with TAK242 (1 µM) or C29 (50 µM), an inhibitor of TLR-4 or TLR-2 signaling, respectively, for 30 min [59,60]. We ensured that no trace endotoxins contributed to the observed responses using rWRS treated with 10 µg/mL of PMB for 1 h at room temperature or heated at 15 min at 100 °C. Furthermore, in HUVECs treated with rWRS, the mRNA expressions of MCP-1, ICAM-1, and VCAM-1 were also determined using RT-qPCR. The sequences of primer sets are presented in Supplementary Table S1, and all primers were purchased from Takara Bio. The mRNA levels were examined using GAPDH mRNA as an internal control. The levels of TNF-α, IL-8, and MCP-1 in the culture supernatants were measured using ELISA kits (Proteintech Group, Inc).

### 4.6. Immunofluorescence Study

We added rWRS to the wells of an eight-part slide culture of HUVECs at 5 µg/mL in serum-free HuMedia-EG2 medium. After 12 h of incubation at 37 °C, the cells were fixed with paraformaldehyde. After washing the cells with PBS and then blocking them with 1% fetal calf serum, they were treated with monoclonal anti-ICAM-1 antibodies (1:200; Abcam, Tokyo, Japan) and anti-VCAM-1 antibodies (1:200; Abcam) overnight at 4 °C. The cells were washed with PBS and then treated with Alexa Fluor 594-conjugated goat anti-rabbit IgG (1:1000; Thermo Fisher Scientific), followed by the staining of the nuclei with DAPI (1:1000; Thermo Fisher Scientific). We analyzed the treated cells using fluorescence microscopy (KEYENCE, Osaka, Japan) [61]. The fluorescence intensity was measured using the image processing software ImageJ.

### 4.7. Expressions of LDLR mRNA

The LDLR mRNA expression was analyzed using the method of Hossain et al. with a slight modification [52]. First, the medium of the differentiated THP-1 cells with PMA for 3 days was changed to serum-free RPMI 16490. The THP-1 cells were then incubated in the medium for 4 h at 37 °C. Thereafter, the cells were treated with rWRS (5 µg/mL) or Pg-LPS (10 µg/mL) in serum-free RPMI 1640 at 37 °C for 3 h. After incubation, the SR-A, LOX-1, CD36, and LDLR mRNA expressions in the THP-1 cells were determined using RT-qPCR with the specific primer sets (Supplementary Table S1).

### 4.8. Statistical Analysis

Statistical analyses were conducted using Student’s *t*-test and analysis of variance with Bonferroni’s post-test using MacTKV3 software (Esumi. Co., Ltd., Tokyo, Japan). All values were expressed as the mean ± standard deviation. We considered probability (*p*) values of <0.05 and <0.01 as statistically significant.

## 5. Conclusions

WRS secreted by host cells following infection with *P. gingivalis* induced the expression of inflammatory cytokines, chemokines, and adhesion molecules in macrophages and HUVECs. These factors can trigger the recruitment and inflammatory response of inflammatory macrophages, neutrophils, and white blood cells in blood vessels. In addition, WRS upregulates LDLR and oxLDLR expression, which may induce the formation of foam cells from macrophages and cause atherosclerosis. In fact, *P. gingivalis* is frequently detected in atherosclerotic plaques [30,31]. The sustained release of WRS from host cells following *P. gingivalis* infection in this study suggested a new pathogenic mechanism in atherosclerosis. Regarding diabetes, which is the most studied systemic disease associated with periodontitis, many reports have stated that dental treatment of periodontitis leads to improvements in diabetes [4]. Therefore, the elimination of unwanted physiological activities of these host factors by periodontal treatment could represent a new therapeutic strategy for various systemic inflammatory diseases.

## Data Availability

All data generated and/or analyzed during this study are included in this article.

## Supplementary Materials

The following supporting information can be downloaded at: https://www.mdpi.com/article/10.3390/pathogens10121648/s1, Table S1: Primers applied for quantitative RT-PCR.
