# Supplementary material for: Tryptophanyl tRNA Synthetase from Human Macrophages Infected by Porphyromonas gingivalis Induces a Proinflammatory Response Associated with Atherosclerosis
Source: Pathogens. 2021 Dec 20;10(12):1648. doi: 10.3390/pathogens10121648 (PMC8708850; doi:10.3390/pathogens10121648)
Supplement: Supplementary file 1 [file pathogens-10-01648-s001.zip › pathogens-1500710-supplementary.pdf]

Supplementary Table S1 Primers applied for quantitative RT-PCR

Gene symbol		Sequence (5'-3')	Product size (bp)
WRS	sense	gacaagctcgagcagatcagga	98
	antisense	gcgatcaagggctgcagaa	
TNF-alpha	sense	ctgcctgctgcactttggag	132
	antisense	acatgggctacaggcttgtcact	
IL-6	sense	gccagagctgtgcagatgag	112
	antisense	tcagcaggctggcatttg	
IL-8	sense	gtgcagagggttgtggagaagttt	179
	antisense	accaggaatcttgtattgcatctgg	
CXCL12	sense	gagccaacgtcaagcatctcaa	108
	antisense	ttagcttcgggtcaatgcacac	
CCL2	sense	cttctgtgcctgctgctcata	166
	antisense	ctttgggacacttgctgctg	
ICAM-1	sense	tcacggagctcccagtcctaa	128
	antisense	aaaggcaggttggccaatga	
VCAM-1	sense	cgaaaggcccagttgaagga	141
	antisense	gagcacgagaagctcaggagaaa	
LDLR	sense	agacagtgacagcctccgtcag	139
	antisense	cgcatttacgtgctccgaaac	
MSR1	sense	ttctgtctcaaaccctggctgtaa	142
	antisense	cagacatcccaaacaggctgataa	
LOX-1	sense	acaagatgaagcctgcgaat	213
	antisense	gctgagtaaggttcgcttgg	
CD36	sense	aaagtcactgcgacatgattaatgg	131
	antisense	aacgtcggattcaaatacagcatag	
